# Supplementary material for: Resolving the full spectrum of human genome variation using Linked-Reads
Source: Genome Res. 2019 Apr;29(4):635–45. doi: 10.1101/gr.234443.118 (PMC6442396; doi:10.1101/gr.234443.118)
Supplement: Supplemental Material [file supp_gr.234443.118_Supplemental_longranger-2.2.zip › longranger-2.2.tar.bz2!/lr2.2-public/lib/stan/cmdstan/stan/doc/api/html/scaled__inv__chi__square_8hpp_source.html]

Stan: src/stan/prob/distributions/univariate/continuous/scaled\_inv\_chi\_square.hpp Source File


|  |  |
| --- | --- |
|  | Stan  2.5.0  probability, sampling & optimization |


- Main Page
- Related Pages
- Namespaces
- Classes
- Files

- File List
- File Members

All Classes Namespaces Files Functions Variables Typedefs Enumerations Enumerator Friends Macros Pages

- src
- stan
- prob
- distributions
- univariate
- continuous

scaled\_inv\_chi\_square.hpp

Go to the documentation of this file.

1 #ifndef STAN\_\_PROB\_\_DISTRIBUTIONS\_\_UNIVARIATE\_\_CONTINUOUS\_\_SCALED\_INV\_CHI\_SQUARE\_HPP

2 #define STAN\_\_PROB\_\_DISTRIBUTIONS\_\_UNIVARIATE\_\_CONTINUOUS\_\_SCALED\_INV\_CHI\_SQUARE\_HPP

3

4 #include <boost/random/chi\_squared\_distribution.hpp>

5 #include <boost/random/variate\_generator.hpp>

6

7 #include <stan/agrad/partials\_vari.hpp>

8 #include <stan/math/error\_handling.hpp>

9 #include <stan/math/constants.hpp>

10 #include <stan/math/functions/square.hpp>

11 #include <stan/math/functions/multiply\_log.hpp>

12 #include <stan/math/functions/value\_of.hpp>

13 #include <stan/meta/traits.hpp>

14 #include <stan/prob/constants.hpp>

15 #include <stan/prob/traits.hpp>

16 #include <stan/prob/internal\_math.hpp>

17

18 namespace stan {

19

20  namespace prob {

21

41  template <bool propto,

42  typename T\_y, typename T\_dof, typename T\_scale>

43  typename return\_type<T\_y,T\_dof,T\_scale>::type

44  scaled\_inv\_chi\_square\_log(const T\_y& y, const T\_dof& nu, const T\_scale& s) {

45  static const char\* function

46  = "stan::prob::scaled\_inv\_chi\_square\_log(%1%)";

47

48  using stan::math::check\_positive\_finite;

49  using stan::math::check\_not\_nan;

50  using stan::math::check\_consistent\_sizes;

51  using stan::math::value\_of;

52

53  // check if any vectors are zero length

54  if (!(stan::length(y)

55  && stan::length(nu)

56  && stan::length(s)))

57  return 0.0;

58

59  double logp(0.0);

60  check\_not\_nan(function, y, "Random variable", &logp);

61  check\_positive\_finite(function, nu, "Degrees of freedom parameter",

62  &logp);

63  check\_positive\_finite(function, s, "Scale parameter", &logp);

64  check\_consistent\_sizes(function,

65  y,nu,s,

66  "Random variable",

67  "Degrees of freedom parameter",

68  "Scale parameter",

69  &logp);

70

71  // check if no variables are involved and prop-to

72  if (!include\_summand<propto,T\_y,T\_dof,T\_scale>::value)

73  return 0.0;

74

75  VectorView<const T\_y> y\_vec(y);

76  VectorView<const T\_dof> nu\_vec(nu);

77  VectorView<const T\_scale> s\_vec(s);

78  size\_t N = max\_size(y, nu, s);

79

80  for (size\_t n = 0; n < N; n++) {

81  if (value\_of(y\_vec[n]) <= 0)

82  return LOG\_ZERO;

83  }

84

85  using boost::math::lgamma;

86  using boost::math::digamma;

87  using stan::math::multiply\_log;

88  using stan::math::square;

89

90  DoubleVectorView<include\_summand<propto,T\_dof,T\_y,T\_scale>::value,

91  is\_vector<T\_dof>::value> half\_nu(length(nu));

92  for (size\_t i = 0; i < length(nu); i++)

93  if (include\_summand<propto,T\_dof,T\_y,T\_scale>::value)

94  half\_nu[i] = 0.5 \* value\_of(nu\_vec[i]);

95

96  DoubleVectorView<include\_summand<propto,T\_dof,T\_y>::value,

97  is\_vector<T\_y>::value> log\_y(length(y));

98  for (size\_t i = 0; i < length(y); i++)

99  if (include\_summand<propto,T\_dof,T\_y>::value)

100  log\_y[i] = log(value\_of(y\_vec[i]));

101

102  DoubleVectorView<include\_summand<propto,T\_dof,T\_y,T\_scale>::value,

103  is\_vector<T\_y>::value> inv\_y(length(y));

104  for (size\_t i = 0; i < length(y); i++)

105  if (include\_summand<propto,T\_dof,T\_y,T\_scale>::value)

106  inv\_y[i] = 1.0 / value\_of(y\_vec[i]);

107

108  DoubleVectorView<include\_summand<propto,T\_dof,T\_scale>::value,

109  is\_vector<T\_scale>::value> log\_s(length(s));

110  for (size\_t i = 0; i < length(s); i++)

111  if (include\_summand<propto,T\_dof,T\_scale>::value)

112  log\_s[i] = log(value\_of(s\_vec[i]));

113

114  DoubleVectorView<include\_summand<propto,T\_dof>::value,

115  is\_vector<T\_dof>::value> log\_half\_nu(length(nu));

116  DoubleVectorView<include\_summand<propto,T\_dof>::value,

117  is\_vector<T\_dof>::value> lgamma\_half\_nu(length(nu));

118  DoubleVectorView<!is\_constant\_struct<T\_dof>::value,

119  is\_vector<T\_dof>::value> digamma\_half\_nu\_over\_two(length(nu));

120  for (size\_t i = 0; i < length(nu); i++) {

121  if (include\_summand<propto,T\_dof>::value)

122  lgamma\_half\_nu[i] = lgamma(half\_nu[i]);

123  if (include\_summand<propto,T\_dof>::value)

124  log\_half\_nu[i] = log(half\_nu[i]);

125  if (!is\_constant\_struct<T\_dof>::value)

126  digamma\_half\_nu\_over\_two[i] = digamma(half\_nu[i]) \* 0.5;

127  }

128

129  agrad::OperandsAndPartials<T\_y,T\_dof,T\_scale>

130  operands\_and\_partials(y, nu, s);

131  for (size\_t n = 0; n < N; n++) {

132  const double s\_dbl = value\_of(s\_vec[n]);

133  const double nu\_dbl = value\_of(nu\_vec[n]);

134  if (include\_summand<propto,T\_dof>::value)

135  logp += half\_nu[n] \* log\_half\_nu[n] - lgamma\_half\_nu[n];

136  if (include\_summand<propto,T\_dof,T\_scale>::value)

137  logp += nu\_dbl \* log\_s[n];

138  if (include\_summand<propto,T\_dof,T\_y>::value)

139  logp -= (half\_nu[n]+1.0) \* log\_y[n];

140  if (include\_summand<propto,T\_dof,T\_y,T\_scale>::value)

141  logp -= half\_nu[n] \* s\_dbl\*s\_dbl \* inv\_y[n];

142

143  if (!is\_constant\_struct<T\_y>::value) {

144  operands\_and\_partials.d\_x1[n]

145  += -(half\_nu[n] + 1.0) \* inv\_y[n]

146  + half\_nu[n] \* s\_dbl\*s\_dbl \* inv\_y[n]\*inv\_y[n];

147  }

148  if (!is\_constant\_struct<T\_dof>::value) {

149  operands\_and\_partials.d\_x2[n]

150  += 0.5 \* log\_half\_nu[n] + 0.5

151  - digamma\_half\_nu\_over\_two[n]

152  + log\_s[n]

153  - 0.5 \* log\_y[n]

154  - 0.5\* s\_dbl\*s\_dbl \* inv\_y[n];

155  }

156  if (!is\_constant\_struct<T\_scale>::value) {

157  operands\_and\_partials.d\_x3[n]

158  += nu\_dbl / s\_dbl - nu\_dbl \* inv\_y[n] \* s\_dbl;

159  }

160  }

161  return operands\_and\_partials.to\_var(logp);

162  }

163

164  template <typename T\_y, typename T\_dof, typename T\_scale>

165  inline

166  typename return\_type<T\_y,T\_dof,T\_scale>::type

167  scaled\_inv\_chi\_square\_log(const T\_y& y, const T\_dof& nu, const T\_scale& s) {

168  return scaled\_inv\_chi\_square\_log<false>(y,nu,s);

169  }

170

185  template <typename T\_y, typename T\_dof, typename T\_scale>

186  typename return\_type<T\_y, T\_dof, T\_scale>::type

187  scaled\_inv\_chi\_square\_cdf(const T\_y& y, const T\_dof& nu,

188  const T\_scale& s) {

189  // Size checks

190  if (!(stan::length(y) && stan::length(nu) && stan::length(s)))

191  return 1.0;

192

193  static const char\* function

194  = "stan::prob::scaled\_inv\_chi\_square\_cdf(%1%)";

195

196  using stan::math::check\_positive\_finite;

197  using stan::math::check\_not\_nan;

198  using stan::math::check\_consistent\_sizes;

199  using stan::math::check\_nonnegative;

200  using stan::math::value\_of;

201

202  double P(1.0);

203

204  check\_not\_nan(function, y, "Random variable", &P);

205  check\_nonnegative(function, y, "Random variable", &P);

206  check\_positive\_finite(function, nu, "Degrees of freedom parameter", &P);

207  check\_positive\_finite(function, s, "Scale parameter", &P);

208  check\_consistent\_sizes(function, y, nu, s,

209  "Random variable",

210  "Degrees of freedom parameter",

211  "Scale parameter",

212  &P);

213

214  // Wrap arguments in vectors

215  VectorView<const T\_y> y\_vec(y);

216  VectorView<const T\_dof> nu\_vec(nu);

217  VectorView<const T\_scale> s\_vec(s);

218  size\_t N = max\_size(y, nu, s);

219

220  agrad::OperandsAndPartials<T\_y, T\_dof, T\_scale>

221  operands\_and\_partials(y, nu, s);

222

223  // Explicit return for extreme values

224  // The gradients are technically ill-defined, but treated as zero

225

226  for (size\_t i = 0; i < stan::length(y); i++) {

227  if (value\_of(y\_vec[i]) == 0)

228  return operands\_and\_partials.to\_var(0.0);

229  }

230

231  // Compute CDF and its gradients

232  using boost::math::gamma\_p\_derivative;

233  using boost::math::gamma\_q;

234  using boost::math::digamma;

235  using boost::math::tgamma;

236

237  // Cache a few expensive function calls if nu is a parameter

238  DoubleVectorView<!is\_constant\_struct<T\_dof>::value,

239  is\_vector<T\_dof>::value> gamma\_vec(stan::length(nu));

240  DoubleVectorView<!is\_constant\_struct<T\_dof>::value,

241  is\_vector<T\_dof>::value> digamma\_vec(stan::length(nu));

242

243  if (!is\_constant\_struct<T\_dof>::value) {

244  for (size\_t i = 0; i < stan::length(nu); i++) {

245  const double half\_nu\_dbl = 0.5 \* value\_of(nu\_vec[i]);

246  gamma\_vec[i] = tgamma(half\_nu\_dbl);

247  digamma\_vec[i] = digamma(half\_nu\_dbl);

248  }

249  }

250

251  // Compute vectorized CDF and gradient

252  for (size\_t n = 0; n < N; n++) {

253

254  // Explicit results for extreme values

255  // The gradients are technically ill-defined, but treated as zero

256  if (value\_of(y\_vec[n]) == std::numeric\_limits<double>::infinity()) {

257  continue;

258  }

259

260  // Pull out values

261  const double y\_dbl = value\_of(y\_vec[n]);

262  const double y\_inv\_dbl = 1.0 / y\_dbl;

263  const double half\_nu\_dbl = 0.5 \* value\_of(nu\_vec[n]);

264  const double s\_dbl = value\_of(s\_vec[n]);

265  const double half\_s2\_overx\_dbl = 0.5 \* s\_dbl \* s\_dbl \* y\_inv\_dbl;

266  const double half\_nu\_s2\_overx\_dbl

267  = 2.0 \* half\_nu\_dbl \* half\_s2\_overx\_dbl;

268

269  // Compute

270  const double Pn = gamma\_q(half\_nu\_dbl, half\_nu\_s2\_overx\_dbl);

271

272  P \*= Pn;

273

274  if (!is\_constant\_struct<T\_y>::value)

275  operands\_and\_partials.d\_x1[n]

276  += half\_nu\_s2\_overx\_dbl \* y\_inv\_dbl

277  \* gamma\_p\_derivative(half\_nu\_dbl, half\_nu\_s2\_overx\_dbl) / Pn;

278

279  if (!is\_constant\_struct<T\_dof>::value)

280  operands\_and\_partials.d\_x2[n]

281  += (0.5 \* stan::math::gradRegIncGamma(half\_nu\_dbl,

282  half\_nu\_s2\_overx\_dbl,

283  gamma\_vec[n], digamma\_vec[n])

284  - half\_s2\_overx\_dbl

285  \* gamma\_p\_derivative(half\_nu\_dbl, half\_nu\_s2\_overx\_dbl) )

286  / Pn;

287

288  if (!is\_constant\_struct<T\_scale>::value)

289  operands\_and\_partials.d\_x3[n]

290  += - 2.0 \* half\_nu\_dbl \* s\_dbl \* y\_inv\_dbl

291  \* gamma\_p\_derivative(half\_nu\_dbl, half\_nu\_s2\_overx\_dbl) / Pn;

292

293  }

294

295  if (!is\_constant\_struct<T\_y>::value)

296  for(size\_t n = 0; n < stan::length(y); ++n)

297  operands\_and\_partials.d\_x1[n] \*= P;

298  if (!is\_constant\_struct<T\_dof>::value)

299  for(size\_t n = 0; n < stan::length(nu); ++n)

300  operands\_and\_partials.d\_x2[n] \*= P;

301  if (!is\_constant\_struct<T\_scale>::value)

302  for(size\_t n = 0; n < stan::length(s); ++n)

303  operands\_and\_partials.d\_x3[n] \*= P;

304

305  return operands\_and\_partials.to\_var(P);

306  }

307

308  template <typename T\_y, typename T\_dof, typename T\_scale>

309  typename return\_type<T\_y, T\_dof, T\_scale>::type

310  scaled\_inv\_chi\_square\_cdf\_log(const T\_y& y, const T\_dof& nu,

311  const T\_scale& s) {

312  // Size checks

313  if (!(stan::length(y) && stan::length(nu) && stan::length(s)))

314  return 0.0;

315

316  static const char\* function

317  = "stan::prob::scaled\_inv\_chi\_square\_cdf\_log(%1%)";

318

319  using stan::math::check\_positive\_finite;

320  using stan::math::check\_not\_nan;

321  using stan::math::check\_consistent\_sizes;

322  using stan::math::check\_nonnegative;

323  using stan::math::value\_of;

324

325  double P(0.0);

326

327  check\_not\_nan(function, y, "Random variable", &P);

328  check\_nonnegative(function, y, "Random variable", &P);

329  check\_positive\_finite(function, nu, "Degrees of freedom parameter", &P);

330  check\_positive\_finite(function, s, "Scale parameter", &P);

331  check\_consistent\_sizes(function, y, nu, s,

332  "Random variable",

333  "Degrees of freedom parameter",

334  "Scale parameter",

335  &P);

336

337  // Wrap arguments in vectors

338  VectorView<const T\_y> y\_vec(y);

339  VectorView<const T\_dof> nu\_vec(nu);

340  VectorView<const T\_scale> s\_vec(s);

341  size\_t N = max\_size(y, nu, s);

342

343  agrad::OperandsAndPartials<T\_y, T\_dof, T\_scale>

344  operands\_and\_partials(y, nu, s);

345

346  // Explicit return for extreme values

347  // The gradients are technically ill-defined, but treated as zero

348  for (size\_t i = 0; i < stan::length(y); i++) {

349  if (value\_of(y\_vec[i]) == 0)

350  return operands\_and\_partials.to\_var(stan::math::negative\_infinity());

351  }

352

353  // Compute cdf\_log and its gradients

354  using boost::math::gamma\_p\_derivative;

355  using boost::math::gamma\_q;

356  using boost::math::digamma;

357  using boost::math::tgamma;

358

359  // Cache a few expensive function calls if nu is a parameter

360  DoubleVectorView<!is\_constant\_struct<T\_dof>::value,

361  is\_vector<T\_dof>::value> gamma\_vec(stan::length(nu));

362  DoubleVectorView<!is\_constant\_struct<T\_dof>::value,

363  is\_vector<T\_dof>::value> digamma\_vec(stan::length(nu));

364

365  if (!is\_constant\_struct<T\_dof>::value) {

366  for (size\_t i = 0; i < stan::length(nu); i++) {

367  const double half\_nu\_dbl = 0.5 \* value\_of(nu\_vec[i]);

368  gamma\_vec[i] = tgamma(half\_nu\_dbl);

369  digamma\_vec[i] = digamma(half\_nu\_dbl);

370  }

371  }

372

373  // Compute vectorized cdf\_log and gradient

374  for (size\_t n = 0; n < N; n++) {

375

376  // Explicit results for extreme values

377  // The gradients are technically ill-defined, but treated as zero

378  if (value\_of(y\_vec[n]) == std::numeric\_limits<double>::infinity()) {

379  continue;

380  }

381

382  // Pull out values

383  const double y\_dbl = value\_of(y\_vec[n]);

384  const double y\_inv\_dbl = 1.0 / y\_dbl;

385  const double half\_nu\_dbl = 0.5 \* value\_of(nu\_vec[n]);

386  const double s\_dbl = value\_of(s\_vec[n]);

387  const double half\_s2\_overx\_dbl = 0.5 \* s\_dbl \* s\_dbl \* y\_inv\_dbl;

388  const double half\_nu\_s2\_overx\_dbl

389  = 2.0 \* half\_nu\_dbl \* half\_s2\_overx\_dbl;

390

391  // Compute

392  const double Pn = gamma\_q(half\_nu\_dbl, half\_nu\_s2\_overx\_dbl);

393

394  P += log(Pn);

395

396  if (!is\_constant\_struct<T\_y>::value)

397  operands\_and\_partials.d\_x1[n]

398  += half\_nu\_s2\_overx\_dbl \* y\_inv\_dbl

399  \* gamma\_p\_derivative(half\_nu\_dbl, half\_nu\_s2\_overx\_dbl) / Pn;

400  if (!is\_constant\_struct<T\_dof>::value)

401  operands\_and\_partials.d\_x2[n]

402  += (0.5 \* stan::math::gradRegIncGamma(half\_nu\_dbl,

403  half\_nu\_s2\_overx\_dbl,

404  gamma\_vec[n], digamma\_vec[n])

405  - half\_s2\_overx\_dbl

406  \* gamma\_p\_derivative(half\_nu\_dbl, half\_nu\_s2\_overx\_dbl) )

407  / Pn;

408  if (!is\_constant\_struct<T\_scale>::value)

409  operands\_and\_partials.d\_x3[n]

410  += - 2.0 \* half\_nu\_dbl \* s\_dbl \* y\_inv\_dbl

411  \* gamma\_p\_derivative(half\_nu\_dbl, half\_nu\_s2\_overx\_dbl) / Pn;

412  }

413

414  return operands\_and\_partials.to\_var(P);

415  }

416

417  template <typename T\_y, typename T\_dof, typename T\_scale>

418  typename return\_type<T\_y, T\_dof, T\_scale>::type

419  scaled\_inv\_chi\_square\_ccdf\_log(const T\_y& y, const T\_dof& nu,

420  const T\_scale& s) {

421  // Size checks

422  if (!(stan::length(y) && stan::length(nu) && stan::length(s)))

423  return 0.0;

424

425  static const char\* function

426  = "stan::prob::scaled\_inv\_chi\_square\_ccdf\_log(%1%)";

427

428  using stan::math::check\_positive\_finite;

429  using stan::math::check\_not\_nan;

430  using stan::math::check\_consistent\_sizes;

431  using stan::math::check\_nonnegative;

432  using stan::math::value\_of;

433

434  double P(0.0);

435

436  check\_not\_nan(function, y, "Random variable", &P);

437  check\_nonnegative(function, y, "Random variable", &P);

438  check\_positive\_finite(function, nu, "Degrees of freedom parameter", &P);

439  check\_positive\_finite(function, s, "Scale parameter", &P);

440  check\_consistent\_sizes(function, y, nu, s,

441  "Random variable",

442  "Degrees of freedom parameter",

443  "Scale parameter",

444  &P);

445

446  // Wrap arguments in vectors

447  VectorView<const T\_y> y\_vec(y);

448  VectorView<const T\_dof> nu\_vec(nu);

449  VectorView<const T\_scale> s\_vec(s);

450  size\_t N = max\_size(y, nu, s);

451

452  agrad::OperandsAndPartials<T\_y, T\_dof, T\_scale>

453  operands\_and\_partials(y, nu, s);

454

455  // Explicit return for extreme values

456  // The gradients are technically ill-defined, but treated as zero

457  for (size\_t i = 0; i < stan::length(y); i++) {

458  if (value\_of(y\_vec[i]) == 0)

459  return operands\_and\_partials.to\_var(0.0);

460  }

461

462  // Compute cdf\_log and its gradients

463  using boost::math::gamma\_p\_derivative;

464  using boost::math::gamma\_q;

465  using boost::math::digamma;

466  using boost::math::tgamma;

467

468  // Cache a few expensive function calls if nu is a parameter

469  DoubleVectorView<!is\_constant\_struct<T\_dof>::value,

470  is\_vector<T\_dof>::value> gamma\_vec(stan::length(nu));

471  DoubleVectorView<!is\_constant\_struct<T\_dof>::value,

472  is\_vector<T\_dof>::value> digamma\_vec(stan::length(nu));

473

474  if (!is\_constant\_struct<T\_dof>::value) {

475  for (size\_t i = 0; i < stan::length(nu); i++) {

476  const double half\_nu\_dbl = 0.5 \* value\_of(nu\_vec[i]);

477  gamma\_vec[i] = tgamma(half\_nu\_dbl);

478  digamma\_vec[i] = digamma(half\_nu\_dbl);

479  }

480  }

481

482  // Compute vectorized cdf\_log and gradient

483  for (size\_t n = 0; n < N; n++) {

484

485  // Explicit results for extreme values

486  // The gradients are technically ill-defined, but treated as zero

487  if (value\_of(y\_vec[n]) == std::numeric\_limits<double>::infinity()) {

488  return operands\_and\_partials.to\_var(stan::math::negative\_infinity());

489  }

490

491  // Pull out values

492  const double y\_dbl = value\_of(y\_vec[n]);

493  const double y\_inv\_dbl = 1.0 / y\_dbl;

494  const double half\_nu\_dbl = 0.5 \* value\_of(nu\_vec[n]);

495  const double s\_dbl = value\_of(s\_vec[n]);

496  const double half\_s2\_overx\_dbl = 0.5 \* s\_dbl \* s\_dbl \* y\_inv\_dbl;

497  const double half\_nu\_s2\_overx\_dbl

498  = 2.0 \* half\_nu\_dbl \* half\_s2\_overx\_dbl;

499

500  // Compute

501  const double Pn = 1.0 - gamma\_q(half\_nu\_dbl, half\_nu\_s2\_overx\_dbl);

502

503  P += log(Pn);

504

505  if (!is\_constant\_struct<T\_y>::value)

506  operands\_and\_partials.d\_x1[n]

507  -= half\_nu\_s2\_overx\_dbl \* y\_inv\_dbl

508  \* gamma\_p\_derivative(half\_nu\_dbl, half\_nu\_s2\_overx\_dbl) / Pn;

509  if (!is\_constant\_struct<T\_dof>::value)

510  operands\_and\_partials.d\_x2[n]

511  -= (0.5 \* stan::math::gradRegIncGamma(half\_nu\_dbl,

512  half\_nu\_s2\_overx\_dbl,

513  gamma\_vec[n], digamma\_vec[n])

514  - half\_s2\_overx\_dbl

515  \* gamma\_p\_derivative(half\_nu\_dbl, half\_nu\_s2\_overx\_dbl) )

516  / Pn;

517  if (!is\_constant\_struct<T\_scale>::value)

518  operands\_and\_partials.d\_x3[n]

519  += 2.0 \* half\_nu\_dbl \* s\_dbl \* y\_inv\_dbl

520  \* gamma\_p\_derivative(half\_nu\_dbl, half\_nu\_s2\_overx\_dbl) / Pn;

521  }

522

523  return operands\_and\_partials.to\_var(P);

524  }

525

526  template <class RNG>

527  inline double

528  scaled\_inv\_chi\_square\_rng(const double nu,

529  const double s,

530  RNG& rng) {

531  using boost::variate\_generator;

532  using boost::random::chi\_squared\_distribution;

533

534  static const char\* function

535  = "stan::prob::scaled\_inv\_chi\_square\_rng(%1%)";

536

537  using stan::math::check\_positive\_finite;

538

539  check\_positive\_finite(function, nu, "Degrees of freedom parameter",

540  (double\*)0);

541  check\_positive\_finite(function, s, "Scale parameter", (double\*)0);

542

543  variate\_generator<RNG&, chi\_squared\_distribution<> >

544  chi\_square\_rng(rng, chi\_squared\_distribution<>(nu));

545  return nu \* s / chi\_square\_rng();

546  }

547  }

548 }

549 #endif

550

stan::math::square

T square(const T x)

Return the square of the specified argument.

**Definition:** square.hpp:22

multiply\_log.hpp

traits.hpp

stan::agrad::tgamma

fvar< T > tgamma(const fvar< T > &x)

**Definition:** tgamma.hpp:15

stan::agrad::OperandsAndPartials::to\_var

T\_return\_type to\_var(double logp)

**Definition:** partials\_vari.hpp:138

stan::math::check\_positive\_finite

bool check\_positive\_finite(const char \*function, const T\_y &y, const char \*name, T\_result \*result)

**Definition:** check\_positive\_finite.hpp:12

error\_handling.hpp

stan::math::multiply\_log

boost::math::tools::promote\_args< T\_a, T\_b >::type multiply\_log(const T\_a a, const T\_b b)

Calculated the value of the first argument times log of the second argument while behaving properly w...

**Definition:** multiply\_log.hpp:51

square.hpp

stan::length

size\_t length(const T &)

**Definition:** traits.hpp:159

stan::DoubleVectorView

DoubleVectorView allocates double values to be used as intermediate values.

**Definition:** traits.hpp:358

partials\_vari.hpp

stan::agrad::value\_of

T value\_of(const fvar< T > &v)

Return the value of the specified variable.

**Definition:** value\_of.hpp:16

constants.hpp

stan::prob::scaled\_inv\_chi\_square\_ccdf\_log

return\_type< T\_y, T\_dof, T\_scale >::type scaled\_inv\_chi\_square\_ccdf\_log(const T\_y &y, const T\_dof &nu, const T\_scale &s)

**Definition:** scaled\_inv\_chi\_square.hpp:419

stan::is\_vector

**Definition:** traits.hpp:83

stan::agrad::lgamma

fvar< T > lgamma(const fvar< T > &x)

**Definition:** lgamma.hpp:15

stan::agrad::OperandsAndPartials

A variable implementation that stores operands and derivatives with respect to the variable...

**Definition:** partials\_vari.hpp:76

traits.hpp

stan::return\_type::type

boost::math::tools::promote\_args< typename scalar\_type< T1 >::type, typename scalar\_type< T2 >::type, typename scalar\_type< T3 >::type, typename scalar\_type< T4 >::type, typename scalar\_type< T5 >::type, typename scalar\_type< T6 >::type >::type type

**Definition:** traits.hpp:406

stan::is\_constant\_struct

Metaprogram to determine if a type has a base scalar type that can be assigned to type double...

**Definition:** traits.hpp:57

stan::math::value\_of

double value\_of(const T x)

Return the value of the specified scalar argument converted to a double value.

**Definition:** value\_of.hpp:24

stan::prob::include\_summand

Template metaprogram to calculate whether a summand needs to be included in a proportional (log) prob...

**Definition:** traits.hpp:35

stan::agrad::gamma\_q

fvar< T > gamma\_q(const fvar< T > &x1, const fvar< T > &x2)

**Definition:** gamma\_q.hpp:15

stan::prob::scaled\_inv\_chi\_square\_cdf

return\_type< T\_y, T\_dof, T\_scale >::type scaled\_inv\_chi\_square\_cdf(const T\_y &y, const T\_dof &nu, const T\_scale &s)

The CDF of a scaled inverse chi-squared density for y with the specified degrees of freedom parameter...

**Definition:** scaled\_inv\_chi\_square.hpp:187

stan::agrad::OperandsAndPartials::d\_x2

VectorView< double \*, is\_vector< T2 >::value, is\_constant\_struct< T2 >::value > d\_x2

**Definition:** partials\_vari.hpp:83

stan::prob::scaled\_inv\_chi\_square\_log

return\_type< T\_y, T\_dof, T\_scale >::type scaled\_inv\_chi\_square\_log(const T\_y &y, const T\_dof &nu, const T\_scale &s)

The log of a scaled inverse chi-squared density for y with the specified degrees of freedom parameter...

**Definition:** scaled\_inv\_chi\_square.hpp:44

stan::math::gradRegIncGamma

double gradRegIncGamma(double a, double z, double g, double dig, double precision=1e-6)

**Definition:** internal\_math.hpp:181

stan::math::check\_nonnegative

bool check\_nonnegative(const char \*function, const T\_y &y, const char \*name, T\_result \*result)

**Definition:** check\_nonnegative.hpp:52

stan::math::check\_consistent\_sizes

bool check\_consistent\_sizes(const char \*function, const T1 &x1, const T2 &x2, const char \*name1, const char \*name2, T\_result \*result)

**Definition:** check\_consistent\_sizes.hpp:13

stan::max\_size

size\_t max\_size(const T1 &x1, const T2 &x2)

**Definition:** traits.hpp:191

internal\_math.hpp

stan::prob::scaled\_inv\_chi\_square\_cdf\_log

return\_type< T\_y, T\_dof, T\_scale >::type scaled\_inv\_chi\_square\_cdf\_log(const T\_y &y, const T\_dof &nu, const T\_scale &s)

**Definition:** scaled\_inv\_chi\_square.hpp:310

stan::math::check\_not\_nan

bool check\_not\_nan(const char \*function, const T\_y &y, const char \*name, T\_result \*result)

Checks if the variable y is nan.

**Definition:** check\_not\_nan.hpp:56

stan::agrad::digamma

fvar< T > digamma(const fvar< T > &x)

**Definition:** digamma.hpp:16

value\_of.hpp

stan::agrad::OperandsAndPartials::d\_x1

VectorView< double \*, is\_vector< T1 >::value, is\_constant\_struct< T1 >::value > d\_x1

**Definition:** partials\_vari.hpp:82

stan::agrad::OperandsAndPartials::d\_x3

VectorView< double \*, is\_vector< T3 >::value, is\_constant\_struct< T3 >::value > d\_x3

**Definition:** partials\_vari.hpp:84

constants.hpp

stan::agrad::log

fvar< T > log(const fvar< T > &x)

**Definition:** log.hpp:15

stan::prob::scaled\_inv\_chi\_square\_rng

double scaled\_inv\_chi\_square\_rng(const double nu, const double s, RNG &rng)

**Definition:** scaled\_inv\_chi\_square.hpp:528

stan::VectorView

VectorView is a template metaprogram that takes its argument and allows it to be used like a vector...

**Definition:** traits.hpp:275

stan::math::negative\_infinity

double negative\_infinity()

Return negative infinity.

**Definition:** constants.hpp:123

stan::prob::chi\_square\_rng

double chi\_square\_rng(const double nu, RNG &rng)

**Definition:** chi\_square.hpp:442


---

[ Stan Home Page ]

*© 2011–2014,
Stan Development Team.*
